# Emergence of a KPC-90 Variant that Confers Resistance to Ceftazidime-Avibactam in an ST463 Carbapenem-Resistant Pseudomonas aeruginosa Strain

**DOI:** 10.1128/spectrum.01869-21

**Published:** 2022-01-12

**Authors:** Yuexing Tu, Dairong Wang, Yiwei Zhu, Jiayan Li, Yan Jiang, Wenhao Wu, Xi Li, Hua Zhou

**Affiliations:** a Department of Rehabilitation Medicine, Zhejiang Provincial People’s Hospital, People’s Hospital of Hangzhou Medical College, Hangzhou, Zhejiang, China; b Blood Center of Zhejiang Province, Hangzhou, Zhejiang, China; c Department of Critical Care Medicine, Renji Hospital affiliated with Shanghai Jiao Tong University School of Medicine, Shanghai, China; d Department of Infectious Diseases, Sir Run Run Shaw Hospital, Zhejiang University School of Medicine, Hangzhou, Zhejiang, China; e Centre of Laboratory Medicine, Zhejiang Provincial People’s Hospital, People’s Hospital of Hangzhou Medical College, Hangzhou, Zhejiang, China; f Department of Respiratory and Critical Care Medicine, the First Affiliated Hospital, Zhejiang University School of Medicine, Hangzhou, Zhejiang, China; National University Hospital

**Keywords:** ceftazidime-avibactam, resistance, CRPA, KPC-90, ST463

## Abstract

Carbapenem-resistant Pseudomonas aeruginosa (CRPA) has become a serious challenge in the clinic. Recently, the prevalence of CRPA isolates carrying the *bla*_KPC-2_ gene has been increasing in China. Ceftazidime-avibactam (CZA) has shown good efficacy against large portions of KPC-2-producing CRPA strains. However, with the increasing usage of this drug, CZA resistance in CRPA strains has been reported. Here, we reported for the first time that resistance of the ST463 CRPA strain to CZA was caused by a novel variant in the KPC gene that arose after CZA exposure. The CRPA strain PA2207 is a carbapenem- and CZA-resistant strain that harbors a mutated *bla*_KPC_ gene, named *bla*_KPC-90_. Cloning and expression of *bla*_KPC-90_ in Escherichia coli DH5α revealed that KPC-90 led to a 64-fold increase in the MIC value of CZA. Conjugation experiments further confirmed that *bla*_KPC-90_ was located on a conjugative plasmid. Whole-genome sequencing analysis showed that this plasmid had high sequence similarity to a previously reported novel *bla*_KPC-2_-harboring plasmid in a clinical P. aeruginosa strain isolated in China. In addition, overexpression of an efflux pump (MexXY-OprM) might be associated with the CZA resistance phenotype, as determined by reverse transcription-quantitative PCR and efflux pump inhibition experiments. For the first time, we reported a KPC variant, KPC-90, in a clinical ST463 CRPA strain with CZA resistance that was mediated by a 2 amino acid insertion outside the KPC omega-loop region. Our study further highlights that diverse KPC variants that mediate CZA resistance have emerged in the CRPA strain. Furthermore, KPC-90 mutation combined with efflux pump overexpression resulted in a high level of resistance to CZA in the PA2207 isolate. Effective surveillance should be conducted to prevent CZA resistance from spreading in the CRPA strain.

**IMPORTANCE** For the first time, we reported a KPC variant, KPC-90, in a clinical ST463 CRPA strain with CZA resistance. CZA resistance was mediated by a 2 amino acid insertion outside the KPC omega-loop region in CRPA. Our study further emphasized that CZA resistance caused by *bla*_KPC_ gene mutation could be selected in CRPA after CZA therapy. Considering the widespread presence of the ST463 CRPA strain in China, clinicians should pay attention to the risk of the development of CZA resistance in CRPA strains under treatment pressure.

## INTRODUCTION

Carbapenem-resistant Pseudomonas aeruginosa (CRPA) infection is commonly associated with high morbidity and mortality and is becoming a serious challenge in the clinic due to the limited treatment options (1). The common mechanisms underlying resistance in CRPA include the acquisition of carbapenemases, the inactivation of the outer membrane protein OprD, the overexpression of efflux pumps, and/or the hyperexpression of chromosomally encoded AmpC-lactamases (2). Notably, recent studies, including our unpublished data, demonstrated that the production of carbapenemases, especially the carbapenemase KPC-2, has become the main mechanism underlying resistance in CRPA in China (3, 4). In addition, sequence type 463 (ST463) is the dominant lineage responsible for the dissemination of KPC-2-producing CRPA (3, 4). The current treatment options for KPC-2-producing CRPA infections are very limited. Ceftazidime-avibactam (CZA) is a novel β-lactam/β-lactamase inhibitor combination that inactivates several classes of β-lactamases, such as Amp-C enzymes, extended spectrum β-lLactamases (ESBLs) and Klebsiella pneumoniae carbapenemase (KPC) enzymes (5). Many *in vitro* studies have demonstrated that CZA shows good efficacy against large portions of MDR/XDR P. aeruginosa strains (6). However, with the increasing usage of this drug, CZA resistance has been reported. In CRPA strains, in addition to MBL-positive strains, P. aeruginosa’s resistance to CZA is mainly associated with AmpC enzyme mutations that lead to hyperproduction, structural modification, and efflux pump and ESBL enzyme mutations (2, 7, 8). In *Enterobacteriaceae*, mutations in the KPC enzyme are mainly associated with CZA resistance. However, the resistance of P. aeruginosa to CZA caused by mutations in the KPC enzyme has not been reported. With the widespread of KPC-2-producing CRPA strains in China, CZA resistance will further limit the treatment options for CRPA infections. Moreover, CZA-resistant strains appear to have a higher mortality rate (almost 40%) (9).

In this study, we used genomic and molecular genetic approaches for the first time to show that KPC-90, a 2 amino acid insertion outside the KPC-2 omega-loop region, mediated CZA resistance in CRPA. Furthermore, we found that a high level of resistance to ceftazidime-avibactam in CRPA was correlated with KPC enzyme mutation and efflux pump overexpression.

## RESULTS AND DISCUSSION

### Clinical microbiological characteristics.

**FIG 1:**
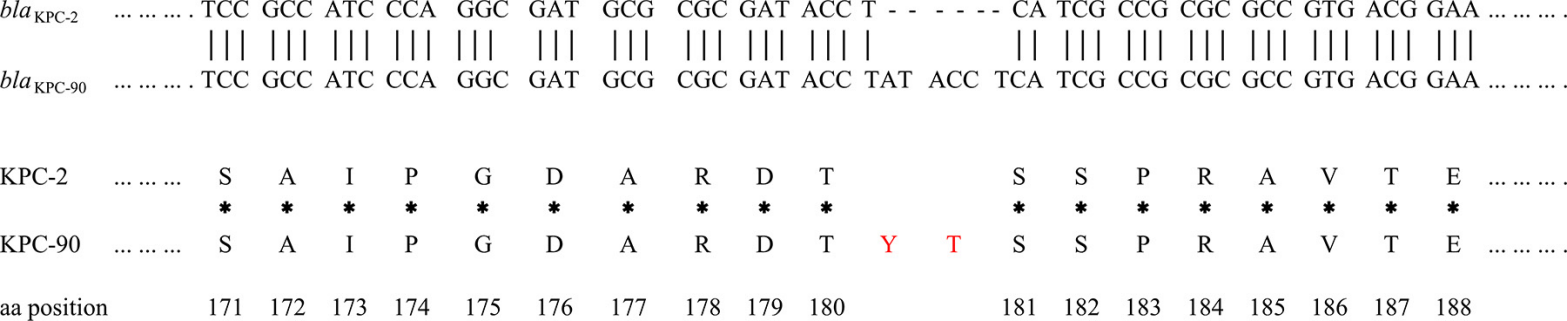
Amplicon alignments between *bla*_KPC-90_ and *bla*_KPC-2_ in nucleotide and amino acid (aa) sequences surrounding the mutation. Six nucleotide deletions were identified at the *bla*_KPC-90_ gene compared to *bla*_KPC-2_, which result in amino acid insertions at the 180 and 181 amino acid positions of the KPC-2 protein. The red letters represent mutant amino acids. Dotted line, common sequence; broken line, deletion of six nucleotides; *, common amino acid; bold font, deletion of two amino acids; A, Ala; D, Asp; E, Glu; G, Gly; I, Ile; P, Pro; R, Arg; S, Ser; T, Thr; V, Val; Y, Tyr.

The antimicrobial susceptibility of the PA2207 strain showed that it was resistant to cefepime, ceftazidime, imipenem, meropenem, ceftolozane-tazobactam, and CZA (MIC, 256 mg/liter) but susceptible to amikacin and colistin (Table 1). Notably, the PA2207 strain had a highly resistant phenotype to CZA. CZA resistance is usually involved in mutations in the *bla*_KPC_ gene (10). To clarify the resistance mechanism, the *bla*_KPC_ gene of the PA2207 strain was amplified by PCR and sequenced. A mutated *bla*_KPC_ gene designated *bla*_KPC-90_ in PA2207 was detected and showed a 6 nucleotide insertion at positions between 538 and 539, resulting in a mutated variant with the two amino acid insertions (Tyr-Thr) between amino acids 180 and 181 (Fig. 1). To further understand the clinical microbiological characteristics of the PA2207 isolate, whole-genome sequencing was performed.

**TABLE 1:** Antibiotic susceptibility of the strains used in this study (mg/liter)^*a*^

Strains	C/T	FEP	CAZ	ETP	IPM	MEM	AMK	CIP	TZP	CST	CZA
E. coli DH5α	0.25	<0.125	0.25	<0.125	0.25	<0.125	2	<0.125	0.5	0.125	0.25
E. coli DH5α/pCR2.1^*b*^	0.25	0.5	1	<0.125	0.5	<0.125	2	<0.125	0.5	<0.125	0.25
E. coli DH5α/pKPC-2^*c*^	4	>128	64	64	64	16	4	<0.125	128	0.125	0.25
E. coli DH5α/pKPC-90^*d*^	16	32	>128	0.25	<0.125	<0.125	1	<0.125	1	<0.125	16
P. aeruginosa PA2207	>256	>128	>128	>128	64	128	8	>128	>256	1	512
PAO1/pPA2207_2^*e*^	>256	>128	>128	64	16	8	4	1	16	0.25	16
P. aeruginosa PAO1^RIF^^*f*^	1	2	1	16	4	2	4	0.5	2	0.5	1
E. coli ATCC 25922	0.125	<0.125	0.125	0.008	0.125	0.015	0.5	0.125	1	0.25	0.25

a Avibactam was added at 4 mg/liter. C/T, ceftolozane-tazobactam; FEP, cefepime; CAZ, ceftazidime; ETP, ertapenem; IPM, imipenem; MEM, meropenem; AMK, amikacin; CIP, ciprofloxacin; TEP, piperacillin-tazobactam; CST, colistin; CZA, ceftazidime-avibactam.

b E. coli DH5α/pCR2.1, E. coli DH5α was transformed by expression plasmid pCR2.1-TOPO as a control.

c E. coli DH5α/pKPC-2, E. coli DH5α was transformed by a pKPC-2 plasmid carrying wild-type *bla*_KPC-2_ gene.

d E. coli DH5α/pKPC-90, E. coli DH5α was transformed by a pKPC-90 plasmid carrying *bla*_KPC-90_ gene.

e PAO1/pPA2207_2, P. aeruginosa PAO1^RIF^ was transformed by pPA2207_2 plasmid carrying wild-type *bla*_KPC-90_ gene.

f P. aeruginosa PAO1^RIF^, a spontaneous rifampicin-resistant mutant P. aeruginosa PAO1strain (19).

The whole-genome sequencing data revealed that the strain belonged to the ST463 clonal lineage. ST463 P. aeruginosa has become a potential high-risk clone because it harbors various virulence genes (11). Potential virulence genes, including T3SS effectors (*toxA*, *exoU*, *exoS*, *exoT,* and *exoY*), T6SS effectors (*Vgr1a* and *Vgr1b*), phenazine biosynthetic genes (*phzH* and *phzM* which promote the conversion of phenazine-1-carboxylic acid to Pyocyanin via an exometabolic pathway, and cause serious damage to the human respiratory tract) and adherence factors (*fliC*) were detected in the PA2207 strain. This strain also harbored various resistance genes, such as *erm*(C), *aph(3′)-IIb*, *catB7*, *bla*_PDC-8_, *bla*_OXA-486,_ *and fosA* according to whole-genome sequencing analysis. Expression of the *bla*_PDC-8_ gene resulted in an elevated CZA MIC value (4/4 mg/liter) in the P. aeruginosa strain (12). In addition, a four-nucleotide deletion at position 38 of the OprD-encoding gene was identified, and this deletion caused a frameshift mutation and generated a premature termination codon in the coding sequence.

### KPC-90 is responsible for CZA resistance.

The CZA resistance phenotype was confirmed in E. coli DH5α with genes encoding KPC-2 and KPC-90 were cloned and expressed. In E. coli, transformants with a pCR2.1-TOPO vector carrying the wild-type *bla*_KPC-2_ gene presented resistance to multiple β-lactams, such as ertapenem, imipenem, and meropenem, but susceptibility to CZA (0.25/4 mg/liter). In contrast, cells carrying the *bla*_KPC-90_-containing pCR2.1-TOPO plasmid were susceptible to ertapenem (0.25 mg/liter), imipenem (<0.125 mg/liter), and meropenem (<0.125 mg/liter) but showed increased resistance to CZA (16/4 mg/liter) (Table 1), demonstrating that the *bla*_KPC-90_ gene was able to confer resistance to CZA (64-fold increase in the MIC value) and result in susceptibility to carbapenems. Most KPC mutations conferring CZA resistance can result in carbapenem susceptibility in strains. These mutations include KPC-33 (13), KPC-41 (14), KPC-50 (15), and KPC-82 (16). KPC-90 was also able to produce a phenotype of CZA- resistant, and meropenem- and imipenem-susceptible phenotype. For *Enterobacteriaceae* isolates with carbapenem susceptibility caused by KPC mutations, carbapenems were also considered to be therapeutic options (14, 17). However, in this study, the PA2207 strain harboring the *bla*_KPC-90_ gene had a carbapenem-resistant phenotype due to OprD inactivation, and carbapenems were not a treatment option for P. aeruginosa. Unlike *Enterobacteriaceae*, which harbors KPC mutations that result in the ESBL phenotype, CRPA is commonly resistant to carbapenems due to the inactivation of OprD. CZA resistance caused by KPC mutations further limits treatment options for infections caused by CRPA.

### *bla*_KPC-90_-carrying plasmid.

**FIG 2:**
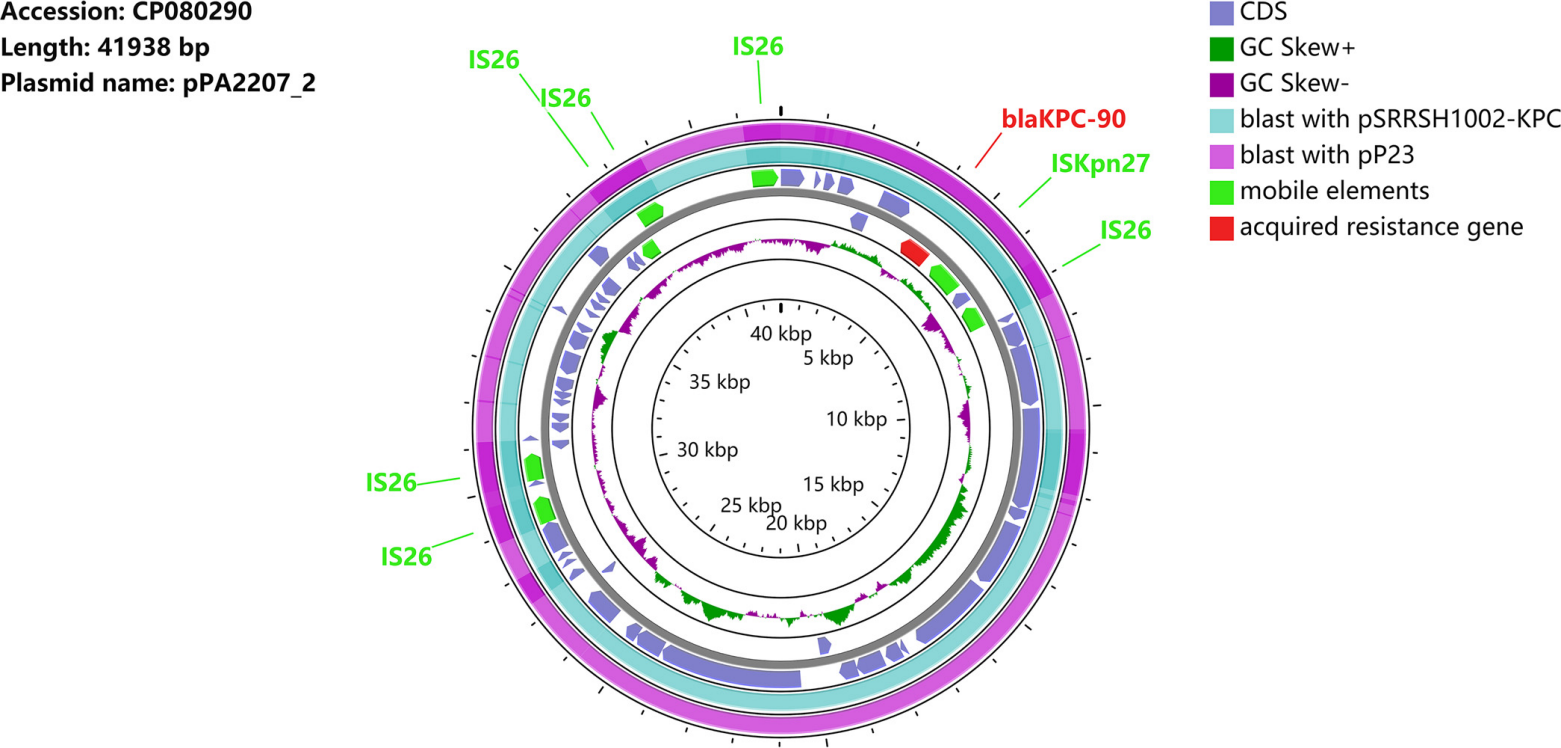
Plasmid analysis of pPA2207_2. Schematic map of plasmid pPA2207_2, this plasmid sequence was compared with plasmids pSRRSH1002-KPC (accession number CP0643988) and pP23 (CP065418).

**FIG 3:**
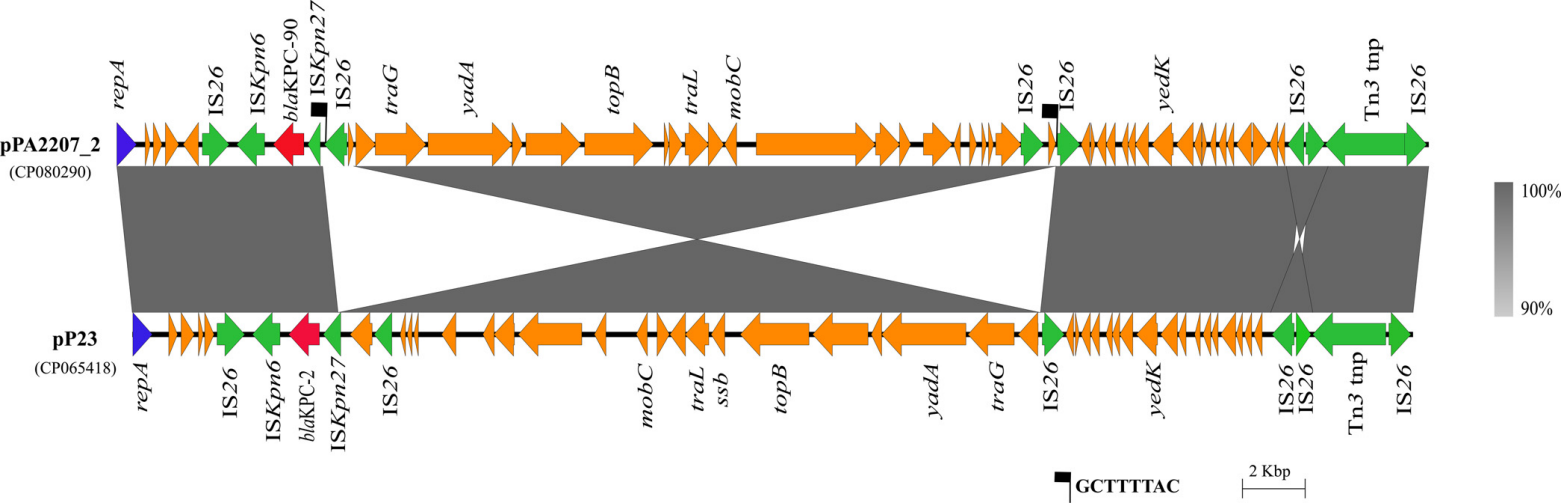
Linear characterization between the plasmid pPA2207_2 (CP080290) and pP23 (CP065418). The gray regions between plasmids indicate nucleotide identity (90 to 100%) by BLASTn. Arrows indicate predicted ORFs. Colored arrows represent open reading frames, with red, yellow, blue, and green representing antibiotic resistance genes, common function genes, replication genes, and mobile elements, respectively.

A conjugation experiment was performed to confirm the transferability of this plasmid. In PAO1/pPA2207_2 transformants carrying the *bla*_KPC-90_ gene, CZA had a MIC of 16/4 mg/liter (a 16-fold increase compared with that in P. aeruginosa PAO1), indicating that the plasmid was able to transfer the CZA-resistant phenotype to the recipient strain (Table 1). The complete plasmid sequence showed that the plasmid was 41,938 bp in length with 58.3% GC content (Fig. 2). The mutant *bla*_KPC-90_ gene was bracketed by IS*26*, IS*kpn27,* and IS*kpn6*, followed by IS*26* genes, and it belonged to the IS*26*-based composite transposon (Fig. 3). There is no other resistance gene in this plasmid. Further sequence alignments revealed that the plasmid sequence showed almost identical nucleotide sequences (99% coverage and 99.99% identity) to those of the CRPA plasmids pSRRSH1002-KPC (accession number CP065418) (18) and pP23 (accession number CP065418) which is a novel *bla*_KPC-2_ gene-containing plasmid isolated from the ST463-type CRPA strain P23 (19). Compared with pPA2207_2 to pP23, a part of the core module was reversed. The fragment between the target on the two IS*26* elements was reversed due to reverse intramolecular replication and translocation, which suggested that two IS*26* copy-side 8-bp sequences (5′-GCTTTTAC-3′) are counter complementary to each other (Fig. 3).

CZA resistance in *Enterobacteriaceae* strains is usually associated with mutations in the *bla*_KPC_ gene (13–16), especially in the omega-loop of the protein (amino acid positions 164 to 179). In contrast to *Enterobacteriaceae*, the resistance of P. aeruginosa to CZA caused by KPC mutations has not been reported. In this study, *bla*_KPC-90_ was demonstrated to be associated with CZA resistance in P. aeruginosa and located in a transportable plasmid. In addition, multiple IS*26* elements that have been proven to undergo frequent intramolecular transposition were found identified in this plasmid. These may also result in the further dissemination of CZA-resistant CRPA bacteria.

### Overexpression of efflux pumps is associated with CZA resistance.

**FIG 4:**
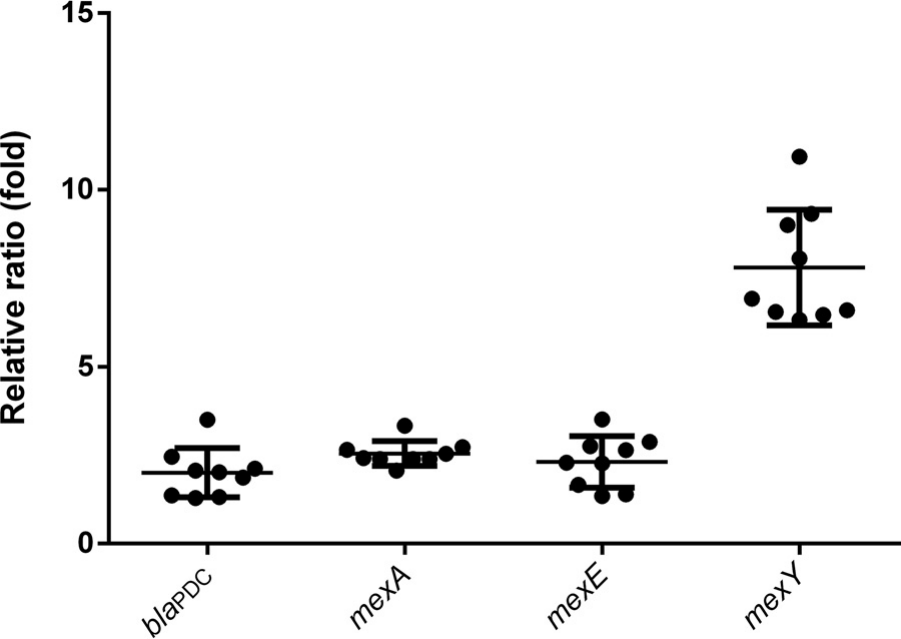
Relative ratio of gene expression of AmpC and efflux pumps. Gene expression was normalized versus the *rpsL* housekeeping gene and expression levels were indicated as a ratio to the expression level in P. aeruginosa strain PA01.

In this study, the P. aeruginosa PA2207 isolate showed high levels of resistance to CZA (MIC, 256 μg/mL), but the transformants had exhibited lower levels of resistance (MIC, 16 μg/mL), indicating that mechanisms other than KPC-90 contributed to this phenotype. Currently, the overexpression of efflux pumps and AmpC enzymes in P. aeruginosa have been demonstrated to be associated with CZA resistance (7, 8). Thus, we quantified the relative expression levels of efflux pumps (MexAB-OprM, MexEF-OprN, and MexXY-OprM) and AmpC enzyme genes to determine whether these factors are associated with resistance to CZA in this isolate. Our results suggest that *mexA*, *mexE,* and *bla*_PDC_ expression were not significantly increased but *mexY* expression was significantly increased (7.799 ± 0.5461-fold) in the PA2207 isolate compared with the susceptible P. aeruginosa PAO1 isolate (Fig. 4), indicating that the MexXY-OprM efflux pump might contribute to CZA resistance.

To confirm this result, efflux pump inhibition assays were further performed. We found that CZA susceptibility in the PA2207 isolate was decreased by 16-fold in the presence of PaβN compared with the absence of PaβN. Overall, the overexpression of efflux pump systems is responsible for the resistance phenotype presented by our isolate.

### Conclusions.

We described, for the first time, a novel KPC variant, KPC-90, that harbors a mutation outside the omega-loop region that arises after CZA exposure and confers resistance to CZA in an ST463-type clinical CRPA isolate. In addition, KPC-90 mutation combined with efflux pump overexpression results in a high level of resistance to CZA in the PA2207 isolate. Notably, CZA resistance was observed in *Enterobacteriaceae* in patients with or without a history of CZA therapy. Our study further emphasized that CZA resistance caused by *bla*_KPC_ gene mutation could be selected for in CRPA after CZA therapy. CZA is currently an important option for the treatment of infections with KPC-2-producing CRPA bacteria. Considering the widespread presence of the ST463 CRPA strain in China, clinicians should pay attention to the risk of the development of CZA resistance in CRPA strains in the context of treatment pressure. The usage of CZA in the clinic should undergo more surveillance, including susceptibility to this drug and *bla*_KPC_ variant that arise after treatment.

## MATERIALS AND METHODS

### Isolate data.

A CZA-resistant CRPA strain, designated PA2207, was recovered from fecal samples from a patient who suffered from leukemia during the process of screening for carbapenem-resistant *Enterobacteriaceae* (CRE) screening. The patient had previously been treated with multiple antimicrobials, including CZA. The strain was identified by the MALDI-TOF MS system (bioMérieux, Marcy l'Etoile, France) and further confirmed by whole-genome sequencing.

### Antimicrobial susceptibility testing.

We performed susceptibility experiments using the broth microdilution method recommended by the Clinical and Laboratory Standards Institute (CLSI). The antimicrobial agents used for susceptibility testing included cefepime (FEP), ceftazidime (CAZ), amikacin (AMK), ciprofloxacin (CIP), meropenem (MEM), imipenem (IPM), colistin (CST), ceftolozane-tazobactam, and CZA. E. coli ATCC 25922 was used as the reference strain. The MIC results were interpreted according to the CLSI guidelines (20), except for colistin where the results were interpreted according to the European Committee on Antimicrobial Susceptibility Testing (EUCAST) guidelines (21).

### Cloning experiments.

Cloning experiments were carried out according to the methods described in a previous study (21). Briefly, the wild-type *bla*_KPC-2_ gene and *bla*_KPC-90_ gene sequences containing the wild promoter were amplified. The purified PCR products were cloned into the pCR2.1-TOPO vector (Invitrogen, Shanghai, China). The recombinant plasmids pKPC-2 and pKPC-90 were both introduced into the E. coli DH5α strain via chemical transformation experiments. Transformants were selected from Luria-Bertani (LB) agar plates supplemented with 50 mg/liter kanamycin, and they were further confirmed by PCR and Sanger sequencing. Primer was listed in Table S1.

### Plasmid conjugation experiments.

Conjugation experiments were performed using a spontaneous rifampicin-resistant mutant of P. aeruginosa PAO1 as the recipient strain (10). P. aeruginosa PA2207 was used as the conjugative donor strain. Transconjugants were isolated on Mueller-Hinton agar plates supplemented with rifampicin (300 μg/mL) and ceftazidime-avibactam (4/4 μg/mL). The presence of the *bla*_KPC-90_ gene in the transconjugants was determined by PCR and Sanger sequencing.

### Reverse transcription-quantitative PCR.

The expression of efflux pumps (MexAB-OprM, MexEF-OprN, and MexXY-OprM) and the AmpC enzyme were quantified according to the method described in our previous study (22). Briefly, total RNA was extracted from bacterial cells in the logarithmic growth phase using an E.Z.N.A. total RNA kit I (Omega Bio-Tek, GA, USA). The relative expression of the efflux pumps and the AmpC enzyme was determined by real-time PCR using the Qiagen QuantiTect SYBR green RT-PCR kit (Qiagen) with a LightCycler 2.0 real-time PCR system. Reactions were repeated in triplicate and normalized to an endogenous reference gene (*rpsL*). For efflux pump and AmpC expression, transcription levels were considered significantly different if at least a 5- or 10-fold difference was observed compared with the expression in P. aeruginosa PAO1, respectively (23). Specific qPCR primer sequences were listed in Table S1.

### Efflux pump inhibition.

The MICs of CZA were determined in the presence and absence of PAβN (TaKaRa Bio Inc., Otsu, Shiga, Japan) at a concentration of 50 μg/mL (24). The isolates were confirmed to overexpress efflux pumps when the MICs in the presence of PAβN were determined to be at least 4-fold lower than the MICs in the absence of PAβN (24). The wild-type P. aeruginosa PAO1 strain was used as the reference strain.

### Genomic DNA extraction and analysis.

Genomic DNA of PA2207 was extracted from PA2207 by using the QIAamp DNA minikit (Qiagen, Valencia, CA, United States). The DNA libraries were prepared by using the Illumina HiSeq X 10 platform (Illumina, San Diego, CA), and a MinION device (Oxford Nanopore Technologies Inc., UK) was used for further genome sequencing according to a previous report (19). The resulting sequence reads were assembled into contigs using CLC Genomics Workbench 10.0. The genome sequence was annotated using The Rapid Annotation using Subsystems Technology (RAST) annotation website server (25).

In addition, we used the ResFinder 4.1 server and MLST 2.1 server (26) to identify the acquired resistance genes (ARGs) and multilocus sequence typing (MLST) of the strain. Virulence genes were analyzed by using BLAST software (SRST2 Toolkit version 0.2.0) (27), and the database of virulence genes at the NCBI. A comparison of the pPA2207_2 plasmid sequence and its related plasmid pP23 sequence was performed with EasyFig v 2.2.3 (28).

### Accession numbers.

The genome sequence of P. aeruginosa PA2207 reported in this study has been deposited in the GenBank nucleotide database under accession no. CP080289 (chromosome of P. aeruginosa PA2207) and CP080290 (plasmid pPA2207_2). In addition, the *bla*_KPC-90_ sequence was deposited in the NCBI database under accession no. MZ570431.
