# Supplementary material for: Emergence of a KPC-90 Variant that Confers Resistance to Ceftazidime-Avibactam in an ST463 Carbapenem-Resistant Pseudomonas aeruginosa Strain
Source: Microbiol Spectr. 2022 Jan 12;10(1):e01869-21. doi: 10.1128/spectrum.01869-21 (PMC8754116; doi:10.1128/spectrum.01869-21)
Supplement: SUPPLEMENTAL FILE 1 — Supplemental material. Download SPECTRUM01869-21_Supp_1_seq9.pdf, PDF file, 0.1 MB [file spectrum01869-21_supp_1_seq9.pdf]

Supplementary Materials

Table S1. Description of primers used in this study.

Primer name	Sequence （5'-3')	reference
RT-mexY-F	TTACCTCCTCCAGCGGC	[1]
RT-mexY-R	GTGAGGCGGGCGTTGTG	
RT-mexA - F	GGCGACAACGCGGCGAAGG	[2]
RT-mexA - R	CCTTCTGCTTGACGCCTTCCTGC	
RT-mexE - F	TCATCCCACTTCTCCTGGCGCTACC	[2]
RT-mexE - R	CGTCCCACTCGTTCAGCGGTTGTTCGATG	
RT-rpsL - F	CGGCACTGCGTAAGGTATGC	[3]
RT-rpsL - R	CGTACTTCGAACGACCCTGCT	
RT-PDC-8_F	AGACCTTCACCGCCACCCT	this study
RT-PDC-8_R	GGATCTGTGCCTGGTCCTTCTG	
KPC-TOPO_F	CCCCAGTCGTCATAACAC	this study
KPC-TOPO_R	TCTACAACCACAGCATTCC	

[1] Hamid R. et al. Role of MexAB-OprM and MexXY-OprM efflux pumps and class 1 integrons in resistance to antibiotics in burn and Intensive Care Unit isolates of Pseudomonas aeruginosa. Journal of Infection and Public Health. 2018, 11: 364-372.

[2] Tomás, M. et al. Efux pumps, OprD porin, AmpC β-lactamase, and multiresistance in Pseudomonas aeruginosa isolates from cystic fbrosis patients. Antimicrobial Agents Chemotherapy. 2010, 54, 2219-2224.

[3] Chalhoub, H. et al. High-level resistance to meropenem in clinical isolates of Pseudomonas aeruginosa in the absence of carbapenemases: role of active efux and porin alterations. International Jouurnal of Antimicrobial Agents. 2016, 48, 740–743.
